# *Daiquiri*: a web-based user interface framework for beamline control and data acquisition

**DOI:** 10.1107/S1600577521009851

**Published:** 2021-10-29

**Authors:** Stuart Fisher, Marcus Oscarsson, Wout De Nolf, Marine Cotte, Jens Meyer

**Affiliations:** a European Synchrotron Radiation Facility, 71 Avenue des Martyrs, 38043 Grenoble, France; b LAMS, CNRS UMR 8220, Sorbonne Universités, Univ Paris 06, 4 Place Jussieu, 75005 Paris, France

**Keywords:** synchrotron beamline control software, graphical user interface, data collection, remote access, *daiquiri*

## Abstract

*Daiquiri* is a web-based user interface (UI) framework for control system monitoring and data acquisition designed for synchrotron beamlines. It provides simple, intuitive and responsive interfaces to control and monitor hardware, launch acquisition sequences and manage associated metadata. *Daiquiri* concerns itself only with the UI layer; it does not provide a scan engine or controls system but can be easily integrated with existing systems.

## Abstract 2

*Daiquiri* is a web-based user interface framework for control system monitoring and data acquisition.

## 1. Introduction

Many synchrotrons make use of some sort of graphical user interface (GUI) to control their beamlines and acquire data. Historically, macromolecular crystallography (MX) has been at the forefront of these developments due to the rigid nature of the experiments involved, many years experience in software development, and the demand for efficiency and automation. This has resulted in the development of software packages such as *MXCuBE(1–3)* (Mueller *et al.*, 2017 ▸; Oscarsson *et al.*, 2019 ▸), co-developed and available at many of the European synchrotrons, as well as *GDA* (and *MXGDA*) (Enderby & Pulford, 2004 ▸) at Diamond Light Source, and *BlueIce* (Stepanov *et al.*, 2011 ▸) at NSLS-II. Synoptic overviews of beamline layouts and control of hardware elements have a long history and include user interfaces (UIs) such as *EDM* and *MEDM* for *EPICS* (Dalesio *et al.*, 1994 ▸), and *Taurus* (Pascual-Izarra *et al.*, 2015 ▸) for the *Tango* control systems (Götz *et al.*, 2020 ▸). Other facilities make use of more generic programs to control their beamlines and accelerators such as *LabView* (Kalkman, 1995 ▸).

At microscopy and imaging beamlines the use of a GUI is essential for efficient observation of the sample, and for selecting regions (ROIs) and points of interest (POIs). At the ESRF, the ID21 beamline pioneered the development of such a GUI 20 years ago. This beamline is dedicated to 2D micro-X-ray fluorescence (µXRF) mapping and micro-X-ray absorption spectroscopy (µXAS) (Cotte *et al.*, 2017 ▸). Typical samples are cells, plant or animal tissue sections, and transversal cross-sections of paintings. They are composed of complex and heterogeneous mixtures of organic and metallic components. Micro-analyses usually aim to identify and localize elements (with µXRF 2D maps), and assess their chemical state (acquisition of µXAS spectra over tens of points or acquisition of µXRF maps at tens of energies). A GUI is essential to observe the samples *in situ*, to define 2D maps both on the visible-light image and on previously recorded X-ray maps, and to select points for µXAS analysis. This is necessary to make the beamline microscope as easy to use as a visible-light or electron microscope, in particular for non-expert users.

The new ESRF source, the so-called Extremely Brilliant Source (EBS) delivered in 2019–2020, is pioneering in terms of flux, with X-ray performances increased by a factor of 100. Under these conditions where data acquisition times are significantly reduced, it is important to develop tools to ensure that the time for setting up the beamline instruments, mounting the samples, identifying ROIs and POIs, and launching acquisition is reduced to the minimum. The optimization of a UI for beamline control and data acquisition is in this respect fundamental. In addition, the ESRF upgrade includes a large campaign of software development, notably the conversion of the control system from *SPEC* to *BLISS* (Guijarro *et al.*, 2018 ▸). This has also helped drive the development of a new GUI at ID21. The main initial specification was the following:

(i) To develop a common framework that could be easily deployed and adapted to as many beamlines as possible.

(ii) To target in priority needs from ID21 and of the other microscopy beamlines.

(iii) To provide the existing tools for sample visualization, ROI and POI definition, but also to further improve the tools for data acquisition (in particular authentication and authorization, action queue and metadata).

(iv) To offer a UI framework that aims to combine both the data acquisition and controls system interfaces.

(v) Finally to make these available via the web, thus having the intrinsic benefit of being automatically suitable for remote access. In this way, the same UI can be used for local and remote access. Many synchrotrons are moving towards more remote access methods and the COVID-19 pandemic has significantly accelerated developments in this area.

Although *MXCuBE3* fulfils many of these requirements, it is targeted specifically towards MX and has a fixed UI layout dedicated to diffraction experiments. It would have been possible to refactor front- and back-end components of *MXCuBE3* to make them more generic but this would have presented considerable difficulties in the context of a world-wide collaboration. Therefore a new framework was designed that would be more generic and applicable to a more diverse range of beamlines in the future. This paper gives a first presentation of the so-called ‘*daiquiri*’ framework, the new UI developed at the ESRF.

## 2. Concepts

*Daiquiri* does not provide a scan engine or a controls system, it provides only the UI layer. Interaction with the scan engine is conducted via actors, scan data are accessible via an interface, and hardware element control and notification makes use of a very thin abstraction layer. These can technically connect to any control system and integration is relatively straightforward. At the present time, adapters are available for *BLISS* and *Tango* objects, and scans are integrated with the *BLISS* scan engine. Connecting to a controls system requires implementing an abstract device, for example a motor, and creating a local implementation for the controls system of interest which tells *daiquiri* how to interact with this object. The scan data interface has functions to retrieve 0, 1 and 2D data and hooks for the underlying controls system to send events to *daiquiri*. This allows a scan engine to return data and send notifications when new data are available, thus allowing *daiquiri* to follow scans in real time.

*Daiquiri* implements a number of high-level concepts in the context of a data acquisition application. These are as follows:

*Authentication and authorization.* Being a web application, *daiquiri* enforces login to know who is accessing the application (users, beamline staff, support staff) and what they are allowed to do. Users must have a valid allocated beam time session to access the interface. Privileges for staff members can be elevated giving access to extra user interfaces, scan types and hardware elements.

*Multi-user, single point of control.* *Daiquiri* allows multiple sessions to be connected simultaneously and implements a fairly common baton style system to enable control of the beamline and avoid multiple users executing actions simultaneously. Only a single user can be in control of the beamline at a time with other users requesting control as needed. Staff can take control of the beamline at any time.

*Actors.* *Daiquiri* decouples itself from any action it executes via actors; these are a simple Python class with a method that can execute any Python code. These are discussed in more detail later.

*Action queue.* *Daiquiri* implements a basic queue system. Actors can be placed into this queue to run sequentially or be executed immediately. The status of the queue is monitored and reported, and items can be promoted and demoted as needed This allows *daiquiri* to perform overnight and un­attended data collection.

*Metadata.* Internally *daiquiri* makes use of the ISPyB database (Delagenière *et al.*, 2011 ▸) to store information about who can login, their associated beamline session information from a User Office system, and what privileges they have, as well as store metadata associated with data collections. In many cases scan engine data and metadata are only available transiently and hence *daiquiri* must store some of this information to provide useful interactive feedback and downstream analysis.

## 3. Implementation

**Figure 1:**
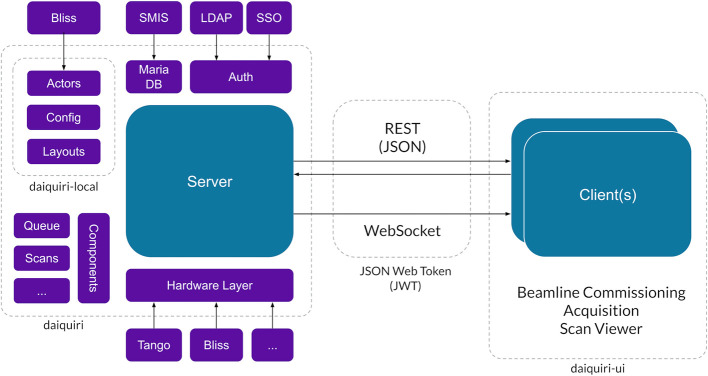
General application architecture.

*Daiquiri* is implemented with a traditional client server methodology to provide clear separation between the UI and associated application programming interface (API). Much inspiration was taken from the *MXCuBE3* project with the intention of producing a more generic framework for acquisition. Many core ideas were also inspired by features and concepts in the *GDA* application. The general application architecture is shown in Fig. 1 ▸ and is implemented in three distinct projects: *daiquiri* (server), *daiquiri-local* (local beamline code and configuration) and *daiquiri-ui* (client).

### 3.1. Server

The server (*daiquiri*) is implemented in Python 3 making use of *Flask*, *Flask-RESTful*, *Flask-SocketIO* and *Marshmallow* to provide a *REST* API and *SocketIO* service for websocket support and event-driven feedback. *Marshmallow* and *apispec* allow for validation of parameters passed to the API and automatic generation of associated documentation. This includes a full description of the *REST* API comprising detailed information about payloads and response types in Swagger/OpenAPI format. The schemas generated by *Marshmallow* are also available to the UI allowing for a single point definition of validation. This avoids mismatched validation between server and client. A full test suite is provided using pytest and a comprehensive continuous integration pipeline provides code style validation, testing, coverage reports and up-to-date API documentation.

Authorization is extensible and there is currently an LDAP adapter. Authorization is controllable on a per beamline basis, restricts access if a user does not have beam time scheduled, and can elevate privileges for staff members.

The server implements the idea of pluggable components. This allows each individual beamline to load only the components relevant to it. Currently these include:

(i) A simple hardware component to monitor and control hardware elements.

(ii) A synoptic view that can show schematic synoptics annotated with hardware values and pop-ups that drill down into groups of hardware elements.

(iii) A console component to allow *daiquiri* to interact with the controls system command line interface.

(iv) A white-listed file editor so that actors, layout files and other beamline configuration files can be created and modified.

(v) A visual light microscope (VLM) component to align samples via a video camera, discussed in more detail later.

(vi) A simple component that executes actors in the context of a defined sample.

(vii) A chat component so users and beamline staff can communicate with each other.

The hardware abstraction layer defines how *daiquiri* should map a particular object from the control system type to its internally defined abstract object model. In the case of *BLISS*, this simply maps the Python attributes. The abstraction layer currently includes objects for basic types such as motors, shutters, cameras and a few other devices. New objects can be easily added and a tutorial is provided in the documentation on how to do this.

For example, the *daiquiri* motor object contains properties such as position, velocity and acceleration, and functions such as move, and rmove (for a relative move). This would be converted into the following JSON by the *REST* API:[Chem scheme1] 
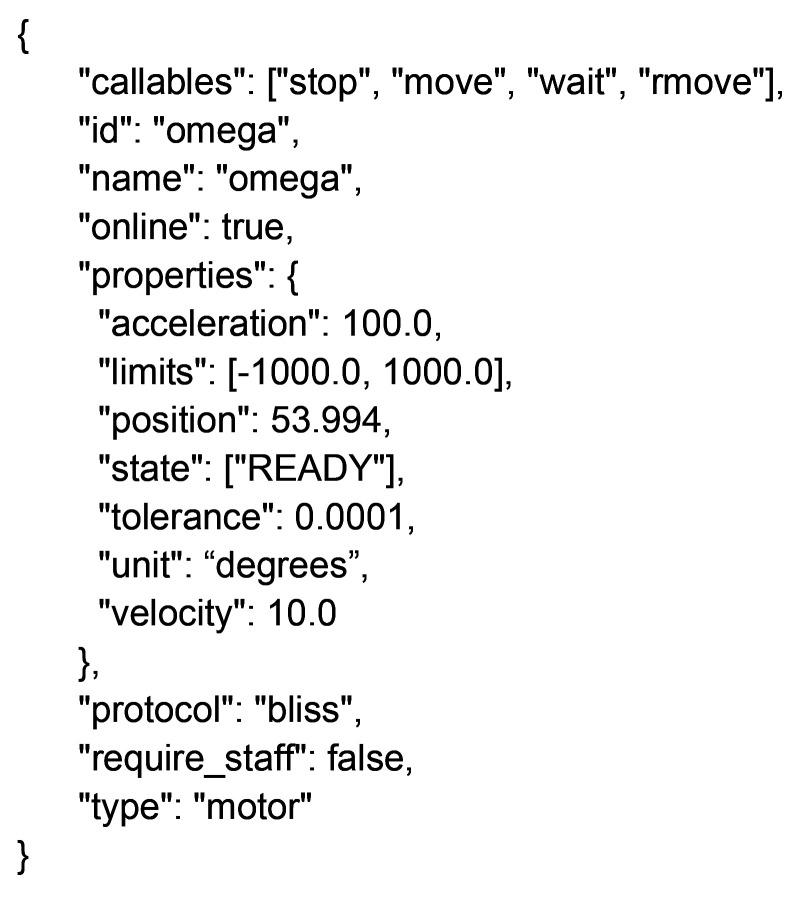
 All hardware objects within *daiquiri* contain the common outer JSON structure, with each individual object implementing its own ‘properties’ and ‘callables’ as per its abstract object.

Local beamline-specific code (for example actors), configuration and layout information is committed into the beamline-specific *daiquiri-local* repository. This is a cookiecutter project that can be instantiated for each beamline and provides a common layout and some sensible default configuration.

### 3.2. Client

The client (*daiquiri-ui*) is implemented in Javascript es6 making use of the popular front-end framework *React* (React, 2013 ▸) with state managed using *Redux* (Redux, 2015 ▸); the application was bootstrapped using *create-react-app* (Create React App, 2016 ▸). Layout is controlled using *react-bootstrap* (React Bootstrap, 2014 ▸; Bootstrap, 2011 ▸) and styled with CSS preprocessor *SASS* (SASS, 2015 ▸). A variety of libraries and helpers allow for rapid development and abstract away some of the complexities of *Redux* and management of asynchronous resources. Testing uses *jest* and *react-testing-library*. A comprehensive continuous integration pipeline provides code style validation, testing, coverage reports and analysis. The client requires no client side configuration and is passed all configuration automatically from the server.

The schemas available from the server API are automatically converted into intuitive forms using *react-jsonschema-form*. This means that developers can define their schema in the server layer and have it automatically transformed into something the user interacts with. This allows controls group staff to develop interfaces to execute, for example, a new scan type actor, rapidly in Python without having to learn the nuances of Javascript.

The UI supports multiple layouts that can be switched between readily. These are generated from a simple YAML structure defined on the server side. The layout manager has basic widgets to organize components into rows, columns and tabs, which map to *bootstrap*’s grid system. Then components can be added to these layout elements, such as a table of scans, plots of scan results, a series of hardware objects, *etc*.

## 4. In use

### 4.1. Classic light microscope

The classic visual-light microscope (VLM) or on-axis viewer (OAV) is an integral part of many beamlines spanning multiple disciplines. It allows for samples to be aligned visually with the incident X-ray beam and scans to be enqueued against positions on the sample. *Daiquiri* provides a comprehensive interface for this concept. However, contrary to traditional implementations, it places the sample at the origin of the canvas rather than the video stream. Traditionally the viewport is fixed to the camera’s field of view. *Daiquiri* allows for the canvas to be zoomed out, and thus allows marking of ROIs and POIs outside of the current field of view, as well as the generation of sample mosaic images where the sample is rastered over a large 2D area, taking a visible image at each point of the mosaic. Using this component a user can navigate around the sample by clicking, mark regions, points and lines, and then enqueue scans against these positions. Resulting maps from scan data (in the case of regions and mapping) can be annotated back onto this view, raw values can be interrogated, and associated spectra can be shown for each pixel. This component could also be readily extended to allow for *n*-click centring.

### 4.2. Monitoring

The UI provides two types of monitoring components. The first, at the top of the screen, provides essential information as to the status of crucial hardware components, such as the ring current, and status of absorbers and shutters. These elements are always visible. The second is a generic hardware monitoring component that allows monitoring and control of, for example, motors, shutters, cameras, *etc*.

### 4.3. Actors

Actors decouple *daiquiri* from other systems. They are simply a class with a method function and an associated schema to define and validate the required parameters. In order to aid rapid development and testing, actors are automatically reloaded both in the server and client on each instantiation. Actors can provide extended validation, for example, when parameters depend on each other, asynchronous validation, for example, revalidation on each keystroke, calculate parameters and report these back to the client, and provide warnings. Code executed in these classes is isolated from the core *daiquiri* code and output from these calls are logged and captured for review to aid debugging.

### 4.4. On the ID21 microscopy beamline

**Figure 2:**
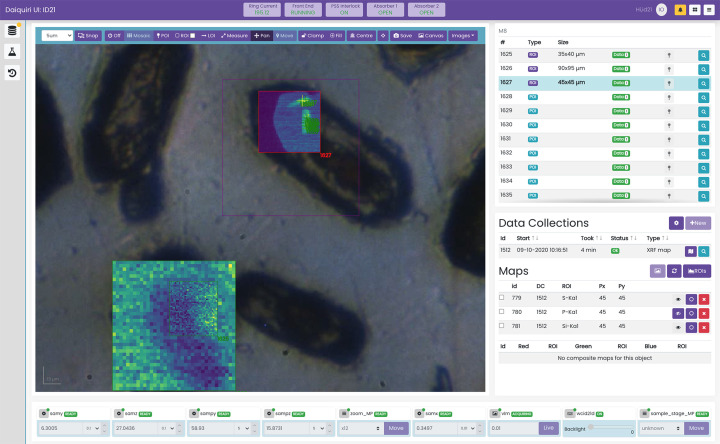
Acquisition interface on ID21, courtesy of H. Moreira. µXRF maps are superimposed on the visible-light image of the sample. On the bottom left µXRF map, a smaller map was selected and acquired with higher resolution. On the top right µXRF map, a series of POIs have been selected for the collection of µXAS spectra.

At ID21, two core interfaces are provided to allow data acquisition and beamline control and alignment. The first, shown in Fig. 2 ▸, uses the VLM component detailed above to allow the user to visualize the sample in real time via a live video stream (frames provided from a LIMA camera, interfaced via *video-streamer-mpeg*, see Section 4.6), to navigate around, mark regions and collect 2D µXRF maps. To the right is the list of currently defined ROIs (in purple) and POIs (in turquoise), and below the data collections conducted and resulting maps on each of these regions. Further below is a general hardware monitoring section to change settings such as the zoom level and move to different sample stage locations.

**Figure 3:**
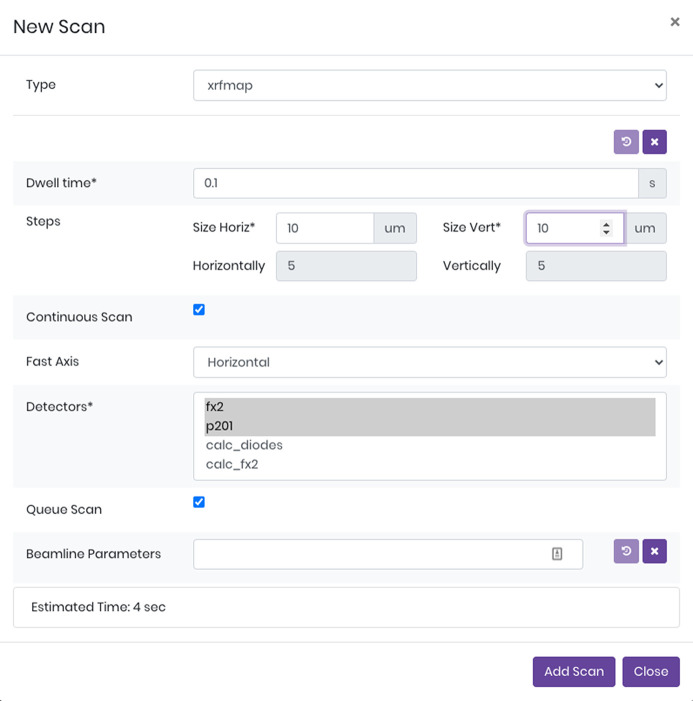
An example new scan dialogue where the user can select the type of action to execute against a particular ROI or POI. In this case an ROI is selected and the user can execute a 2D µXRF map. The form is automatically generated from the relevant actor’s schema and parameter values are validated both in the client and on the server.

Once a region or point is selected a new data collection can be executed against this object. By clicking the ‘New’ button on the Data Collections panel, *daiquiri* will allow the user to select what action to execute based on the available actors defined on the beamline. For example 2D µXRF maps and 2D multispectral µXRF maps can be collected against ROIs, whereas µXAS can be executed against POIs. An example of this dialogue is shown in Fig. 3 ▸ – this is generated automatically from the actor’s validation schema. These actors are committed to the local beamline repository *daiquiri-local*, so can be readily modified and added by beamline staff as experiments evolve. These actions can be placed into *daiquiri*’s queue for sequential execution, so for example a series of µXAS spectra can be collected overnight.

*Daiquiri* has been available to users since the EBS restart (September 2020), and first-time synchrotron users were autonomous in less than an hour.

**Figure 4:**
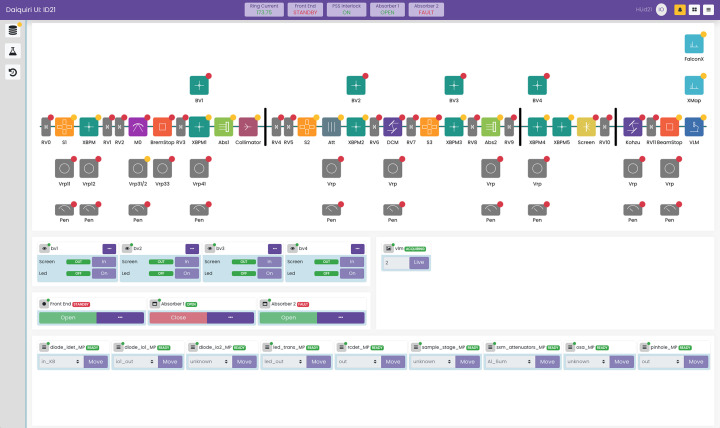
Beamline configuration interface on ID21.

The second interface, shown in Fig. 4 ▸, allows for basic configuration and control of the beamline. There is a synoptic overview of the beamline components along with their status. Beamviewer and front-end components can be controlled, and a variety of other components can be modified. The individual icons within the synoptic view can be configured to show a group of hardware objects that have been defined in the hardware configuration. For example, clicking on a shutter could show the four associated motors.

### 4.5. Containerization

The entire project has been containerized using docker. The container includes a test *BLISS* session, LIMA simulator, *Tango* dummy device, *daiquiri*, and a production-ready minified version of *daiquiri-ui*. The docker container is built nightly and tested using dgoss. This allows *daiquiri* to be easily started for rapid development, demonstration and evaluation. Two images are available: the primary *daiquiri* container and a supporting prepopulated database *daiquiri-testdb*.

### 4.6. Supplementary packages

In addition to *daiquiri* and *daiquiri-ui*, a number of additional packages are available to help stream video, create synoptic schematics and synchronize user office information:

*synoptic svg*: Allows the generation of beamline schematic synoptics from a simple YAML configuration file.

*video-streamer-mpeg*: Takes raw frames from a camera and streams them over websockets using MPEG encoding to provide low-latency real-time video with reasonable compression. The package currently supports LIMA cameras but the frame grabber is implemented via an interface and so is straightforward to extend to other control systems. The package internally uses *FFmpeg* (FFmpeg, 2000 ▸) and can therefore decode and translate frames in RGB, bayer and other formats.

*replicator*: Replicates user office information into the local ISPyB database. It currently implements a SMIS plugin for the ESRF user office system but is readily extensible via an interface. It is designed to be performant so that changes in the user office system can be synchronized on a 15 minute basis.

### 4.7. Third-party integration

Internally *daiquiri* makes use of the some of the *ISPyB* database tables to handle its metadata; this means that if a beamline is making use of *daiquiri* it can also make use of *ISPyB* interfaces such as *SynchWeb* (Fisher *et al.*, 2015 ▸) to manage access rights, monitor beamlines in real time, evaluate beam time usage and review collected data. This also means that if *ISPyB* is used to barcode and ship samples to a facility, *daiquiri* can access the sample information and use this metadata during data collection. For example, chemical information about a sample could help to determine data collection parameters and guide data processing. Then *ISPyB* can be used to ship samples back to users, giving full traceability in a single Laboratory Information Management System (LIMS).

**Figure 5:**
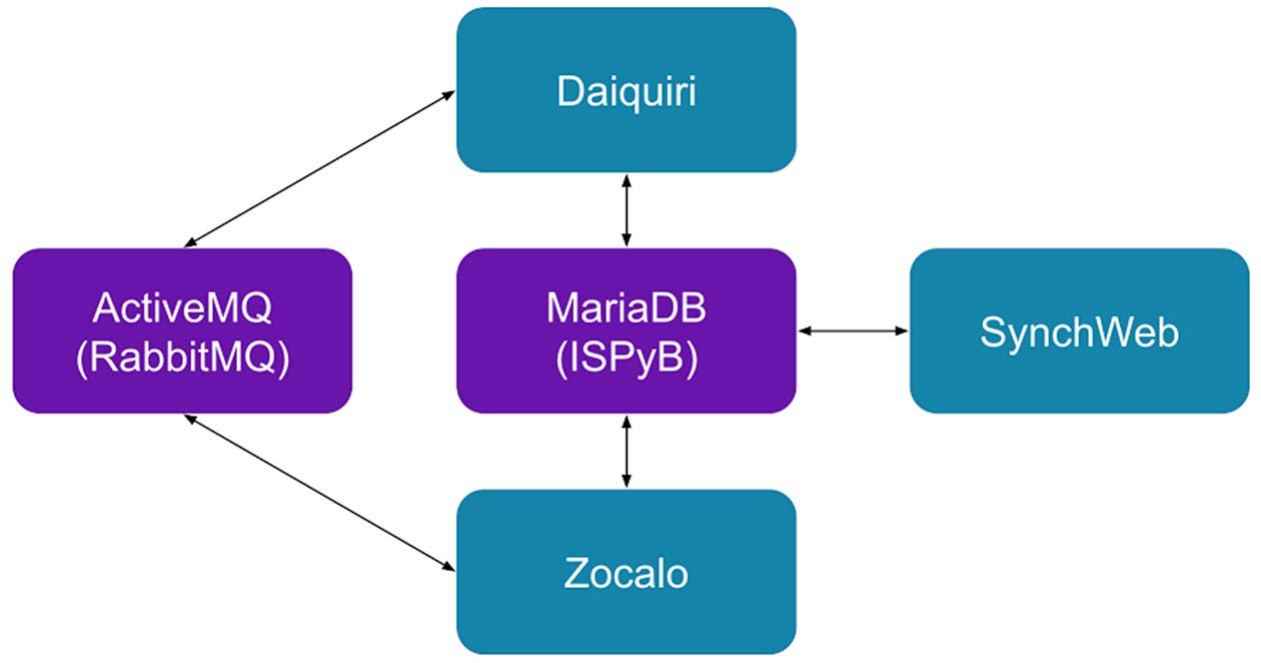
Overall infrastructure and relevant links between *daiquiri* and third-party tools.

*Daiquiri* also has basic support for the *Zocalo* (Gerstel *et al.*, 2020 ▸) automated data processing framework. This means that it can send messages to start processing of data and it can be notified when a processing job is finished. It can then access and display the relevant processing results as these can also be stored in the *ISPyB* database. Fig. 5 ▸ shows the overall infrastructure.

## 5. Conclusions

*Daiquiri* is currently deployed on the X-ray fluorescence mapping and spectroscopy beamline ID21, as well as the BioSAXS beamline BM29 where the server is deployed along with a custom front-end *BSXCuBE3* (Oskarsson *et al.*, 2020 ▸). *Daiquiri* will be extended for BM23 and ID24, both of which conduct mostly EXAFS experiments, the tomography beamline BM18, the diffraction at extreme conditions beamline ID27, and the micro X-ray diffraction beamline ID13. Basic monitoring installations are available on BM05 and ID26. In the future *daiquiri* will be the standard interface by which users and scientists interact with the controls system on many beamlines at the ESRF.

Further information and details on how to try *daiquiri* can be found on the landing page: https://ui.gitlab-pages.esrf.fr/daiquiri-landing.

## 6. Source code

Source code for the relevant projects can be found at https://gitlab.esrf.fr/ui/daiquiri, https://gitlab.esrf.fr/ui/daiquiri-local, https://gitlab.esrf.fr/ui/daiquiri-ui, and documentation at https://ui.gitlab-pages.esrf.fr/daiquiri, https://ui.gitlab-pages.esrf.fr/daiquiri-ui. Docker images can be found on docker hub: https://hub.docker.com/u/esrfbcu.

## References

[bb1] Bootstrap (2011). *Bootstrap – Build fast, responsive sites with Bootstrap*, https://getbootstrap.com/.

[bb2] Cotte, M., Pouyet, E., Salomé, M., Rivard, C., De Nolf, W., Castillo-Michel, H., Fabris, T., Monico, L., Janssens, K., Wang, T., Sciau, P., Verger, L., Cormier, L., Dargaud, O., Brun, E., Bugnazet, D., Fayard, B., Hesse, B., Pradas del Real, A. E., Veronesi, G., Langlois, J., Balcar, N., Vandenberghe, Y., Solé, V. A., Kieffer, J., Barrett, R., Cohen, C., Cornu, C., Baker, R., Gagliardini, E., Papillon, E. & Susini, J. (2017). *J. Anal. At. Spectrom.* **32**, 477–493.

[bb3] Create React App (2016). *Create React App – Set up a modern web app by running one command*, https://create-react-app.dev/.

[bb4] Dalesio, L. R., Hill, J. O., Kraimer, M., Lewis, S., Murray, D., Hunt, S., Watson, W., Clausen, M. & Dalesio, J. (1994). *Nucl. Instrum. Methods Phys. Res. A*, **352**, 179–184.

[bb5] Delagenière, S., Brenchereau, P., Launer, L., Ashton, A. W., Leal, R., Veyrier, S., Gabadinho, J., Gordon, E. J., Jones, S. D., Levik, K. E., McSweeney, S. M., Monaco, S., Nanao, M., Spruce, D., Svensson, O., Walsh, M. A. & Leonard, G. A. (2011). *Bioinformatics*, **27**, 3186–3192. 10.1093/bioinformatics/btr535 21949273

[bb6] Enderby, M. J. & Pulford, B. (2004). *SR Generic Data Acquisition Project Overview, NOBUGS 5*, 18–20 October 2004, PSI, Switzerland.

[bb7] FFmpeg (2000). *FFmpeg – A complete, cross-platform solution to record, convert and stream audio and video*, https://ffmpeg.org/.

[bb8] Fisher, S. J., Levik, K. E., Williams, M. A., Ashton, A. W. & McAuley, K. E. (2015). *J. Appl. Cryst.* **48**, 927–932. 10.1107/S1600576715004847 PMC4453979 26089766

[bb9] Gerstel, M., Ashton, A., Gildea, R., Levik, K. & Winter, G. (2020). *Proceedings of the 17th International Conference on Accelerator and Large Experimental Physics Control Systems (ICALEPCS2019)*, 5–11 October 2019, New York, NY, USA, pp. 1031–1035. WEMPR001.

[bb10] Götz, A., Abeillé, G., Bartolini, M., Bourtembourg, R., Braun, T., Chaize, J.-M., Coutinho, T., Gara, S., Goryl, P., Hardion, V., Joubert, A., Khokhriakov, I., Liszcz, M., Mant, G., Merkulova, O., Moldes, J., Pivetta, L. & Verdier, P. (2020). *Proceedings of the 17th International Conference on Accelerator and Large Experimental Physics Control Systems (ICALEPCS2019)*, 5–11 October 2019, New York, NY, USA, pp. 1234–1239. WEPHA058.

[bb11] Guijarro, M., Beteva, A., Coutinho, T., Dominguez, M.-C., Guilloud, C., Homs, A., Meyer, J., Michel, V., Papillon, E., Perez, M. & Petitdemange, S. (2018). *Proceedings of the 16th International Conference on Accelerator and Large Experimental Physics Control Systems (ICALEPCS2017)*, 8–13 October 2017, Barcelona, Spain, pp. 1060–1066. WEBPL05.

[bb12] Kalkman, C. J. (1995). *J. Clin. Monit. Comput.* **11**, 51–58. 10.1007/BF01627421 7745456

[bb13] Mueller, U., Thunnissen, M., Nan, J., Eguiraun, M., Bolmsten, F., Milàn-Otero, A., Guijarro, M., Oscarsson, M., de Sanctis, D. & Leonard, G. (2017). *Synchrotron Radiat. News*, **30**(1), 22–27.

[bb14] Oscarsson, M., Beteva, A., Flot, D., Gordon, E., Guijarro, M., Leonard, G., McSweeney, S., Monaco, S., Mueller-Dieckmann, C., Nanao, M., Nurizzo, D., Popov, A., von Stetten, D., Svensson, O., Rey-Bakaikoa, V., Chado, I., Chavas, L., Gadea, L., Gourhant, P., Isabet, T., Legrand, P., Savko, M., Sirigu, S., Shepard, W., Thompson, A., Mueller, U., Nan, J., Eguiraun, M., Bolmsten, F., Nardella, A., Milàn-Otero, A., Thunnissen, M., Hellmig, M., Kastner, A., Schmuckermaier, L., Gerlach, M., Feiler, C., Weiss, M. S., Bowler, M. W., Gobbo, A., Papp, G., Sinoir, J., McCarthy, A., Karpics, I., Nikolova, M., Bourenkov, G., Schneider, T., Andreu, J., Cuní, G., Juanhuix, J., Boer, R., Fogh, R., Keller, P., Flensburg, C., Paciorek, W., Vonrhein, C., Bricogne, G. & de Sanctis, D. (2019). *J. Synchrotron Rad.* **26**, 393–405. 10.1107/S1600577519001267 PMC6412183 30855248

[bb15] Oskarsson, M., Beteva, A., De Sanctis, D., Fisher, S., Florial, J. B., Leonard, G., McCarthy, A., Pernot, P. & Tully, M. (2020). *Proceedings of the 17th International Conference on Accelerator and Large Experimental Physics Control Systems (ICALEPCS2019)*, 5–11 October 2019, New York, NY, USA, pp. 1364–1367. WEPHA115.

[bb16] Pascual-Izarra, C., Coutinho, T., Cuní, G., Falcón Torres, C., Fernández-Carreiras, D., Reszela, Z. & Rosanes Siscart, M. (2015). *Proceedings of the 2015 International Conference on Accelerator and Large Experimental Physics Control Systems (ICALEPCS2015)*, 17–23 October 2015, Melbourne, Australia, pp. 1138–1142. THHC3O03.

[bb17] React (2013). *React – A JavaScript library for building user interfaces*, http://reactjs.org/.

[bb18] React Bootstrap (2014). *React Bootstrap – The most popular front-end framework Rebuilt for React*, https://react-bootstrap.github.io/.

[bb19] Redux (2015). *Redux – A Predictable State Container for JS Apps*, https://redux.js.org/.

[bb20] SASS (2015). *CSS with superpowers*, https://sass-lang.com/.

[bb21] Stepanov, S., Makarov, O., Hilgart, M., Pothineni, S. B., Urakhchin, A., Devarapalli, S., Yoder, D., Becker, M., Ogata, C., Sanishvili, R., Venugopalan, N., Smith, J. L. & Fischetti, R. F. (2011). *Acta Cryst.* D**67**, 176–188. 10.1107/S0907444910053916 PMC3046456 21358048

